# Working Memory and Cross-Linguistic Influence on Vocabulary Acquisition

**DOI:** 10.3390/brainsci14080796

**Published:** 2024-08-09

**Authors:** Elizabeth Flores-Salgado, Aldo Falú Gutiérrez-Koyoc

**Affiliations:** Facultad de Lenguas, Benemérita Universidad Autónoma de Puebla, Puebla C.P. 72000, Mexico; gk223462015@alm.buap.mx

**Keywords:** working memory, short-term memory, cross-linguistic influence, typology, close language, distant language

## Abstract

The purpose of this study was to analyze the cross-linguistic influence of previously learned languages and working memory capacities on the vocabulary performance of two different typological languages. The objectives of this study were (1) to compare the working memory capacities of bilingual adults in relation to the vocabulary performance of two different languages never learned by the participants, and (2) to analyze to what extent the typology of previously learned languages influences working memory capacities in relation to the vocabulary performance of French and Nahuatl. A group of 43 Mexican Spanish college students participated in this experimental study. The participants completed a series of working memory tasks in Nahuatl and French. The results showed that working memory capacities were lower in Nahuatl than in French. Thus, a correlation was found between their first and second language and vocabulary performance in French. We can consider the influence of previously learned languages as a significant factor in vocabulary acquisition in accordance with the participants’ working memory capacities.

## 1. Introduction

Vocabulary plays an important role in language and is crucial for the acquisition of a second language. It has been observed that working memory capacities can help learners acquire a second language (L2) more efficiently and achieve a high level of language proficiency [1,2]. According to previous studies [3,4], working memory not only stores information in long-term memory but also integrates new information with known information in long-term memory. Verbal working memory has been linked to L2 grammar acquisition [5,6,7,8], but its role in L2 word acquisition is less clear [9,10,11].

Other factors can influence the acquisition of vocabulary in the L2. One of these factors is the cross-linguistic influence of the first language (L1) or all previously learned languages. Several studies have shown that knowledge of previous linguistic systems can accelerate the acquisition of an additional language [12,13]. It has been found that the cross-linguistic influence on the acquisition of lexis is more pronounced when one of the previous languages is typologically related to the L2 or L3 [12]. Typological closeness refers to the linguistic distance that can exist between one language and another [14]. Thus, there are more lexical similarities and cognate forms in the two languages that are closely related. Therefore, similar languages may facilitate vocabulary acquisition, while different languages may delay this process [15,16,17]. It is assumed that cognates can activate semantically and phonologically similar words in the other language depending on the degree of similarity between L1, L2, or L3. Previous studies have shown that words with similar phonetic and semantic features are stored close together, regardless of whether they belong to the same language or to different languages [18,19,20]. One of the aims of the present study is to investigate the cross-linguistic influence on working memory capacities and vocabulary acquisition of a typologically close language and a typologically distant language. This study focuses on investigating the complex relationship between working memory capacity and vocabulary acquisition in the context of second language learning. Thus, the purpose of this study is to analyze the relationship between verbal working memory and the cross-linguistic influence on vocabulary acquisition.

### 1.1. Working Memory 

Working memory plays a central role in cognitive processes such as language learning. In contrast to long-term memory, which stores information indefinitely, working memory (WM) is a system with limited capacity that is essential for temporarily storing and manipulating information. Previous research [21,22] defines working memory as the ability to store and process small amounts of information simultaneously, which enables cognitive tasks such as problem-solving, decision-making, and language acquisition [23]. Working memory facilitates the integration of new information with knowledge already stored in long-term memory and is therefore crucial for complex cognitive activities [1].

Baddeley’s working memory model identifies three key components: the central executive, the phonological loop, and the visuospatial sketchpad [24]. The central executive, as suggested by previous research [25], acts as a control system that directs attention and coordinates the other components. The phonological loop is specialized in processing and storing auditory information [3,24,26,27,28]. It is crucial for language-related tasks as it enables the rehearsal of verbal information through repetition [3]. This rehearsal process is important for retaining information in short-term memory and transferring it to long-term memory [3].

The visuospatial sketchpad, on the other hand, is responsible for processing visual and spatial information. It allows for the individual to create and manipulate mental images, which is important for tasks involving navigation, geometry, and understanding spatial relationships [3]. Together, these components work dynamically to support a wide range of cognitive activities [24]. An important aspect of this dynamic interaction is the flexibility of the system, as evidenced by another study [3], which allows for it to adapt to different cognitive demands [3]. Cowan emphasizes the dynamic nature of working memory, noting that its capacity is limited but extremely flexible [29]. This flexibility enables the coordination and integration of information from different sources, which is essential for complex cognitive tasks such as problem-solving and decision-making. The interplay between the central executive, the phonological loop, and the visuospatial sketchpad allows for working memory to manage multiple pieces of information. Building on this dynamic nature, Baddeley introduces a fourth component [30].

Baddeley’s model of working memory, developed from his earlier work, introduces a crucial fourth component, the so-called episodic buffer [30]. This component extends the model by integrating information from different domains into coherent episodic representations, facilitating complex cognitive tasks such as comprehension, learning, and problem-solving [30]. Unlike the phonological loop and the visuospatial sketchpad, which are specialized for auditory and visuospatial information, respectively, the episodic buffer serves as a temporary storage system that merges these different types of information with information from long-term memory [30]. While the episodic buffer focuses on integration, other components such as the phonological loop have specific functions that are essential for the maintenance and transfer of information. Finally, rehearsal, a key function of the phonological loop, involves the active repetition of information in order to retain it in memory. This process not only helps with short-term retention but also facilitates the transfer of information into long-term memory so that it can be more easily recalled later.

Understanding the interplay between the components of working memory, including rehearsal, improves our understanding of the cognitive processes involved in language tasks and has implications for L2 vocabulary acquisition. Previous studies suggest that working memory may be related to grammar acquisition, but not to vocabulary [31,32]. Grammar consists of linguistic regularities, in contrast to vocabulary, where there is more variation in word formation. Based on this, it can be said that verbal working memory processes linguistic regularities better. According to Bosma, working memory forms linguistic and non-linguistic schemata through categorization [9]. In the following sections, the role of phonological working memory, the importance of practicing, and the cross-linguistic effects on L2 word acquisition are discussed.

### 1.2. Phonological Working Memory 

The phonological working memory is an important part of our overall working memory. It is responsible for storing and processing the sounds that makeup words, known as phonemes. It is a key component in language learning as it stores and manipulates phonemes for various processes [26]. This temporary storage allows for the learner to perceive and assimilate unfamiliar sounds while learning and practicing the language [33]. In addition, when learning an L2, learners map the new sounds to the L2 using their existing phonological repertoire of the L1 as a frame of reference [34]. This is particularly important when reproducing L2 sounds that have no equivalent in the phonological inventory of the L1. These processes can facilitate mediation but can also lead to challenges. Specifically, a speaker’s L1 phonological forms can negatively impact the perception and production of L2 phonetic structures, especially where the phonetic difference is most pronounced [35]. An efficient phonological working memory plays a crucial role here by helping to manage this interference, inhibiting L1 representations, and facilitating access to unknown L2 forms [36].

The discovery that verbal working memory facilitates learning phonological patterns across different languages is significant for several reasons. Firstly, it provides psycholinguistic support for the presence of these cross-linguistic phonological patterns (Sjölin; Rys; and Taeldeman) as cited in Bosma [15]. In addition, phonological working memory plays a role in recognizing cognates, i.e., words with a common origin and similar meaning in different languages. Studies have shown a correlation between stronger verbal working memory and better performance on tasks involving cognates with phonological transparency [9], where the relationships between sound and writing are consistent [16]. This suggests that phonological working memory aids in interpreting cognates when aligning L1 and L2, especially those with straightforward phonological translations. Furthermore, this relationship between phonological working memory and language processing extends to the crucial role of rehearsal.

### 1.3. Rehearsal in Working Memory 

The relationship between working memory and language learning has attracted considerable attention in the last decade, e.g., [37,38]. Vocabulary acquisition is understood as a process in which people learn and assimilate new words [1]. Cognitive psychology models propose that information is processed through a series of stages. These include the allocation of attention to the input, the rehearsal and processing of the information in working memory, and finally its storage in long-term memory [3]. This means that before storage in long-term memory, there is a learning phase in which the word is held in the focus of attention, processed, and evaluated in the light of existing knowledge or word representations [3].

It is at this stage of learning, located in working memory, that learners are most likely to discover word meanings and create deep memory traces for later recall [24]. Consequently, some form of processing in working memory is considered a necessary prerequisite for vocabulary acquisition. This processing can occur implicitly through exposure to the language, learning new words in context, or making a conscious effort to understand the meaning of unfamiliar words learned in second language classes [39]. Working memory is likely to be an important mediating factor as its main function is to manipulate, monitor, and process information in learning tasks [24]. Learners with greater working memory capacity are therefore at an advantage when acquiring new words and are more likely to retain an extensive and accessible vocabulary [8,40]. This means that it would be beneficial to consider vocabulary learning as a special case in the context of how working memory capacity mediates the acquisition and understanding of word meanings.

In the case of the present study, we aimed to investigate the working memory and vocabulary acquisition of two typologically different L3s (French and Nahuatl) in an attempt to understand the interaction of the L1 and L2 and the process related to L3 acquisition. Therefore, by examining working memory capacity (WMC) and word fluency, this study aimed to provide evidence of the benefits and difficulties of cross-linguistic influence. This research can be seen as relevant to work investigating how different cognitive variables, such as working memory, influence L1 and L2 vocabulary development in bilinguals in the context of language typology.

### 1.4. Cross-Linguistic Influence (CLI) 

In contrast to children who only learn one language, adults who acquire a second language can rely on their existing languages. Previous studies suggest that cross-linguistic influence can have a positive effect on learning [41,42,43,44,45]. Knowledge of two (or more) previous linguistic systems can accelerate the acquisition of a new language. According to Hammarberg, this positive effect is influenced by the following factors: typology, language proficiency, and L2 status [18].

Typological closeness is one of the most influential factors in the L3 acquisition of lexis [12]. Previous research on vocabulary acquisition in the L3 has shown that the typological closeness of two or more languages and their similarities in certain constructions can increase cross-linguistic influence and activate the acquisition of the L3 [9,45]. The competence of the learned languages competes at the moment another language is learned. Thus, the language that is typologically closer to the L3 can become the source of transfer [46]. Therefore, L3 learners transfer existing linguistic knowledge from the typologically closer language [19].

However, typological closeness is not the only source of transfer. The learners’ conscious or unconscious intuitions can also influence what can or cannot be transferred [47]. Transferability or psychotypology is defined as “perceived language distance” [16]. Learners select the linguistic features that are transferable and those that are not. Judgments about perceived distance may not always align with actual typological closeness. Transferability may lead learners to perceive certain structures or words as less typologically similar, even though they belong to the same language family. Previous research has shown that transferability is influenced by proficiency in the target language [17].

Language proficiency is another factor that can influence cross-linguistic transfer. Language learners tend to transfer information from the language they are more proficient in [48]. Likewise, the L1 and L2 play different roles in the acquisition of another language. It has been found that working memory capacity is better in the L1 than in the L2; however, as L2 proficiency increases, working memory capacity is better in the L2. In addition, the gap that exists between L1 and L2 working memory capacity often diminishes [49,50]. Learners may rely more on the L2 than the L1 to acquire vocabulary in the new language [18,48]. The language level of the L2 and L3 can activate previously learned languages. Bardel believes that there is a relationship between the language level of the target language and previously acquired languages [51]. Accordingly, a low language level in the target language L3 activates any learned language with a low level, but a high level in the L3 only activates an L2 with a high level or the L1.

L2 status can also influence the acquisition of a new language. In the initial stages of L3 acquisition, the L2 is activated and takes on a stronger role than the L1 [52]. Amaro, Amaro, and Rothman state that multilingual learners feel more comfortable transferring structures from the L2 to an L3 than to another previously acquired system [53]. In addition, the most recently acquired language may have a stronger influence on the acquisition process than the first language, especially if the L2 and L3 were acquired as foreign languages [54]. Typological closeness can also be a reason for the preference for the L2. Jessner’s research on Indo-European languages shows that L2 patterns are more likely to be transferred by multilingual learners when the L1 is not typologically related to the L3 [55]. L2 status therefore appears to be an important factor in vocabulary acquisition [56].

This study aimed to analyze the cross-linguistic influence of previously learned languages and working memory capacities on the vocabulary performance of two typologically different languages. The objectives of this study were to (1) compare the working memory capacities of bilingual adults in relation to the vocabulary performance of two different languages never learned by the participants, and (2) analyze the extent to which the typology of previously learned languages influences working memory capacities in relation to the vocabulary performance of French and Nahuatl. We hypothesized that the capacity of working memory is lower in L3 Nahuatl compared to L3 French, that the capacity of working memory supports the acquisition of written forms better than the acquisition of cross-linguistic phonological regularities, and that the acquisition of cognates is strongly influenced by the typology of languages, but that all rehearsal contributes to vocabulary acquisition regardless of the language.

## 2. Materials and Methods

In order to analyze the extent of working memory and the cross-linguistic influence on vocabulary acquisition, an experimental design was used in this study. This design has as its main feature the manipulation of a situation to determine the effect that the independent variables (cross-linguistic influence and working memory) can have on the dependent variable (vocabulary acquisition) [57]. The participants, the instruments used, and the procedure are explained in detail in this section.

### 2.1. Participants

Forty-three university students with Mexican Spanish as their first language were recruited from a large Mexican university, 28 women and 15 men. All were from Puebla, Mexico and their ages ranged from 18 to 23. They were second-year students studying English Teaching as a Foreign Language at the Benemérita Universidad Autónoma de Puebla. None of the participants learned French or Nahuatl as a third language. Students accepted into this program are selected by the College’s administrative office. Admission to the program is determined by scores on the entrance exam, which tests math and Spanish proficiency. English proficiency is not assessed and is not a requirement for admission to the program. Students who are accepted attend EFL classes for two hours, five days a week for two years. In order to determine their level of English, the participants took a mock English exam (Mock Cambridge Exam), which corresponded to the lower intermediate level (B1 level according to the guidelines of the Common European Framework). This test covered the components of grammar, reading, writing, and listening comprehension. Accordingly, 65% (*n* = 28) of the participants had a basic proficiency corresponding to A2 level, 25% (*n* = 11) had a lower-intermediate proficiency that placed them at B1 level, and 10% (*n* = 4) had an intermediate proficiency corresponding to B2 level as described in the CEFR framework. In terms of intensity of contact with English, 80% (*n* = 35) of participants spent more than three hours a day with the language. Participants were invited to take part in this study on a voluntary basis and were offered the opportunity to receive additional points for their midterm exams. The moment they agreed, they were asked to fill out and sign a consent form.

### 2.2. Instruments

A comprehensive set of instruments was used to collect data in this study. Some of the instruments were adopted; others were developed specifically for the purpose of this study. They were used in the following order: a background test, a forward digit span test, a backward digit span test, a receptive vocabulary test in French, a receptive vocabulary test in Nahuatl, and a self-assessment questionnaire.

Before describing the instruments, it is necessary to mention that French and Nahuatl were chosen for the following reasons: First, these two languages differ typologically. They belong to two different language families. French belongs to the Romance language family, which is part of the Indo-European language, and Nahuatl belongs to the Yutonahua (utoaztec) family, which is spoken in central and western Mexico. For this reason, French was chosen as the language closest to Spanish, and Nahuatl as the more distant language. Second, Spanish and French have the same origin: they are derived from Latin and belong to the Romance languages. Due to their common Latin root, both languages have some close lexical similarities. On the other hand, Nahuatl belongs to a different language family; however, the Spanish spoken in Mexico was influenced by Nahuatl. Many Nahuatl words have coexisted with Spanish since the conquest of Mexico by Spain (http://www.ethnologue.com accessed on 10 April 2024) [44,58]. Third, English is a language that belongs to the Germanic family; nevertheless, some terms were adopted from Latin in the sixth, seventh, and eighth centuries [59]. Fourth, to account for potential confounding variables, this study controlled for participants’ English language level and their exposure to French and Nahuatl. Therefore, the participants had never learned these two languages. 

#### 2.2.1. Background Questionnaire

In order to know the characteristics of the participants and to ensure that the differences were not due to variables other than those studied, all learners completed a background questionnaire. The background questionnaire consisted of 10 questions in Spanish (the participants’ native language). The questionnaire included the following information: age, gender, mother tongue, additional languages acquired, the number of years they had been learning English, the number of hours they spent practicing English daily, whether they knew French, and whether they knew Nahuatl. To control for the variable of intensity of exposure associated with the acquisition of vocabulary [15], participants were asked the number of years they had learned English and the number of hours they had been exposed to English. Previous studies on cross-linguistic influence have shown that the most recently used other non-target language can have a greater influence on the acquisition of an L3 than the L1, as the lexicon of this language has only recently been assessed and can be easily activated [14,29,60].

#### 2.2.2. Forward Digit Span Test and Backwards Digit Span Test

One of the hypotheses of this study was to test whether verbal working memory supports the acquisition of cross-linguistic phonological and written form regularities. Two tests were used to test this hypothesis: a short-term memory test and a working memory test. These tests make it possible to distinguish between the storage component of verbal working memory and the processing component. Short-term memory was measured using the forward digit span test, as this test only assesses participants’ ability to store a series of digits [61]. The test was chosen in light of the results of previous studies showing that working memory involves both the sequential component (the order in which information is presented) and the representational component (the information itself) [20,62,63]. The sequential component seems to be associated with lexical performance and word learning, and the digit span test appears to be related to this process [38,62,64]. Participants had to repeat sequences of digits in the same order. The sequences became increasingly complex as the task progressed. They began with two digits. The length of the sequences increases until the participant can no longer accurately repeat the numbers. This test is crucial in understanding the capacity and function of working memory, which involves retaining and manipulating information over short periods [3]. The present study employs one sequence per level to streamline the assessment process. This approach aligns with the definition of working memory as the ability to hold and process information briefly [60] and the findings on efficiency of similar studies [65]. On the other hand, working memory was measured with the backwards digit span test, as this test requires considerable processing power from the participants [61]. In this test, participants had to repeat the given digits in reverse order. Similar to the first test, the complexity of the task increased as the test progressed, as one digit was added. Likewise, the length of the sequences increases until the participant can no longer accurately repeat the numbers. To advance to the next level, participants had to recall one more digit.

Before the main study, the forward digit span test and backward digit span test were subjected to a pilot phase in which a small sample of different students took part. The pilot sample consisted of participants from different linguistic backgrounds, including speakers of Spanish, English, and other languages to ensure validity and reliability. English was suggested for both of these tests. It was assumed that all participants were able to count to 10 in English, as all participants were at least A2 level in this language. However, as each test was conducted individually, the participants could decide whether they wanted to rehearse the numbers in their native language or in English. In the end, all students chose to solve the task in English. Before the participants started the test, there was a practice session with two examples. When participants completed a block correctly, they automatically moved on to the next block. They could not continue to the next level if they could not remember one or more digits or omitted digits. At the end of the test, participants had to indicate the last level of difficulty they had reached. Separate scores for digits forward and digits backward were included in the analysis [66].

#### 2.2.3. French and Nahuatl Receptive Vocabulary Test

The purpose of this instrument was to investigate the cross-linguistic influence of previously learned languages and working memory capacities on vocabulary performance in two typologically different languages. The French and Nahuatl receptive vocabulary test was measured with six multiple-choice tasks (three in each language) created specifically for this study. It was developed based on the guidelines of the Peabody Picture Vocabulary Test—Third Edition (PPVTIII) [67]. The PPVTIII is a standardized test used in several studies to measure receptive vocabulary, that is, the understanding of words that are presented orally; working memory capacity; and vocabulary acquisition in children and adults [9,15,68,69]. During the PPVT, the person is shown a series of panels, each with four pictures, the test administrator says a word aloud, and the person being tested must point to it or say which of the four pictures matches the word spoken.

In the present study, the multiple-choice tasks allowed for the systematic assessment of participants’ comprehension of French and Nahuatl words while controlling for possible biases associated with open-ended response formats. There were two versions of the tests, one for French and one for Nahuatl. Each version contained three multiple-choice tasks, and each multiple-choice task contained 30 words. The words were selected in each language based on two criteria: phonology and written form. The first selection was based on the criteria proposed by Bosma et al. [15]. As can be seen in Table 1 and Table 2, the words were divided into three cognate categories, which differed in terms of the degree of cross-linguistic similarity: (1) identical cognates; (2) non-identical cognates, which show simple or non-simple phonological regularity between Spanish, English, and French or Spanish and Nahuatl; (3) non-cognates.

As you can see in Table 1, the French task consisted of Spanish and English cognates. This selection was because the participants were native Spanish speakers, and they were learning English as a second language. The French words were selected on the basis of their phonological similarity between Spanish and English. Nahuatl words, on the other hand, were chosen only by phonetic comparison with Spanish. Because of the way the categories of cognates were defined, it was not possible to have the same proportion of words with identical cognates, non-identical cognates, and non-cognates. Category (2) included most of the items showing cross-linguistic regularity in one, two, or three phonemes, as well as cognates without cross-linguistic regularity. The number of identical cognates (category 1) was difficult to match with non-cognates, especially in Nahuatl.

The written form was the second criterion used to select the words. Three dictionaries were consulted to check the spelling of the 60 target words: (1) the online dictionary of the Royal Academy of the Spanish Language (available at https://dle.rae.es accessed on 10 March 2024), the main academic lexicographical dictionary in Spanish; (2) the online dictionary of the Royal Academy of the French Language (available at https://www.dictionnaire-academie.fr accessed on 10 March 2024), the most representative dictionary in this language; and (3) the online dictionary *Gran Diccionario Nahuátl*, created by the National Autonomous University of Mexico, the National Institute of Anthropology and History, and the National Library of Mexico (available at https://gdn.iib.unam.mx accessed on 10 March 2024).

Table 3 shows that there are words that have the same or four or more similar characters in French, Spanish, and English or in Spanish and Nahuatl. In some cases, some words have the same written form in the languages studied but differ in pronunciation, as can be seen in Table 1.

After selecting the words, a native French speaker and a native Nahuatl speaker were asked to check the phonological and orthographic similarities between the languages studied. At the end of this process, 30 French words and 30 Nahuatl words were selected.

Then, a native French speaker and a native Nahuatl speaker recorded the words. The same 30 selected words of each language were used in the three tasks that were developed. The recordings of the words were inserted as links in Task 1. The multiple-choice tasks were created in Google Forms. This application registered the answers, provided automatic scores, and gave feedback to the participants. The images used in the six multiple-choice tasks were publicly available on the Internet.

One of the objectives of this study was to analyze whether the sound or written form of the word in French or Nahuatl contributes to the identification of the image. In Task 1, participants had to listen to the word’s pronunciation and then look at four pictures on the screen. They then had to select the picture that matched the meaning of the word they listened to. At the end of the task, they received their score and feedback on whether their answer was correct or incorrect.

In Task 2, participants had to look at the word’s written form and see four illustrations on the screen. They then had to choose which illustration matched the written form. As with Task 1, the correct answers to Task 2 were displayed at the end of the test. Feedback was also given indicating the correct answers to the incorrect items. Participants were asked to look at the feedback in detail and note the word and its picture to clarify the meaning.

Task 3 was designed to measure the influence of working memory on vocabulary acquisition. In this task, participants could listen and read the written word. They then had to select the picture representing the meaning of the word they heard and the written form. It is important to mention that the same words were tested in the three tasks. Therefore, Tasks 1 and 2 also served to introduce the words, which were briefly rehearsed in two rounds, followed by Task 3, which assessed the participants’ ability to remember the revised words. The feedback given at the end of Tasks 1 and 2 helped participants to recognize the errors and improve their accuracy for Task 3 [18].

The first version of the six multiple-choice tasks was pilot-tested with forty students from the Benemérita Universidad Autónoma de Puebla. The students were asked to answer the test and add a comment about the clarity of the instructions, the clarity of the audio in Tasks 1 (phonological task) and 3 (combined tasks), and the presentation of the pictures used for each word in the three tasks in the two languages. Two audio recordings in French and one in Nahuatl were replaced as they were not clear. These three items were retested with ten students from the same university. Furthermore, Cronbach’s alpha was calculated to measure the internal consistency of the six multiple-choice tasks of the receptive vocabulary test. These were the results: French I (phonological regularities) α = 0.71, French II (written forms) α = 0.79, French III (combined tasks) α = 0.82, Nahuatl I (phonological regularities) α = 0.70, Nahuatl II (written forms) α = 0.77, Nahuatl III (combined tasks) α = 0.80. These values show that the six tasks have good reliability.

#### 2.2.4. Self-Reflexive Questionnaire

After completing all tasks, a questionnaire based on a Likert scale was immediately filled out to assess the participants’ perception of the role of the previously learned languages in acquiring French and Nahuatl words. This questionnaire was completed in the participant’s native language to corroborate the results and gain a nuanced understanding of the participants’ experiences with memory and vocabulary acquisition. This introspective technique provided a better insight into the mental processes and personal perceptions of the participants and led to better results, as it was carried out immediately after the completion of the tasks [57,70]. Ten questions related to cross-linguistic influence: an assessment of the task (phonological similarity or written form of the word) that helped understand the words in French or Nahuatl, an assessment of the role of previously acquired languages (Spanish and English) in understanding the words in French and Nahuatl, and an assessment of other factors that might influence the understanding of the words. Cronbach’s alpha showed that the internal consistency of the questionnaire items was good, α = 0.80.

### 2.3. Procedure

Forty-three students from two groups agreed to participate in this study and signed a consent form. This study was conducted in two sessions, each lasting two hours, in a computer laboratory. In the first session, the participants’ language level was measured based on the Cambridge Preliminary English Test (level B1). In the second session, all were completed, except for the forward and backward digit span test, which was conducted using another Google application—Google Forms. During the first 20 min, participants completed the forward and backward digit span test, followed by all three French tasks. Afterward, they had a ten-minute break. In the second hour, they worked on the Nahuatl tasks. For Tasks 1 and 3, participants wore headphones to listen to the vocabulary and select the appropriate meaning. Participants progressed at their own pace during this study. After completing all tasks, participants filled out a questionnaire to assess whether hearing the word or seeing the written form was more helpful in recognizing the vocabulary meaning at that moment. They were also asked to rate how much their previously learned languages helped them recognize the meaning of the spoken and written words.

### 2.4. Data Analysis

In order to compare the acquisition of cognates and non-cognates, two identical tests were carried out. The three-factor with replication ANOVA allowed for combining the three tasks (1, 2, and 3) in the two languages (French and Nahuatl) in a single analysis. The three factors were phonological regularities (Task 1), written form (Task 2), and phonological and written form (Task 3). This analysis was replicated in French and Nahuatl with the same participants. The dependent variable was the scores that the participants obtained in the tasks. The independent variables were the two typologically different languages (French and Nahuatl) and the three tasks. A correlational analysis was also conducted to examine the effect of working memory, on the performance of the three tasks in the two studied languages. The scores of the three tasks (separated into cognates and non-cognates) of the two languages (French and Nahuatl) and digit span scores were entered into Pearson correlation analyses. In addition to the quantitative analysis, a qualitative analysis was conducted to analyze the participants’ perceptions of the influence of the previously learned language and the role of working memory in acquiring the vocabulary of a typologically close and a typologically distant language.

## 3. Results

In this section, the results of the present study are presented in relation to the hypotheses focusing on the possible relationship between working memory and the acquisition of cross-linguistic regularities (phonological and written form) in French and Nahuatl.

### 3.1. Descriptive Statistics

The average performance of the participants (mean), the variation in the average performance (standard deviation), and the sample size (*n*) for the receptive vocabulary Tasks 1 (phonological regularities) and 2 (written form) in French and Nahuatl as well as the two cognate categories (identical and non-identical cognates are combined in the cognate category and non-cognates are part of the non-cognate category) are shown in Table 4.

Table 4 shows that there were clear differences between the phonological regularities and the written forms of both cognates and non-cognates in the two languages. Participants performed better on the French written form cognates (M = 16.7; SD = 0.52) and the Nahuatl written form cognates (M = 15.5; SD = 1.48) than on the French phonological cognates (M = 14.5; SD = 1.96) and the Nahuatl phonological cognates (M = 14.7; SD = 1.89). The worst performance was on Nahuatl phonological non-cognates (M = 3.6; SD = 1.89) and the French phonological non-cognates (M = 6; SD = 2.38). Based on these results, it can be said that the participants performed better on the written forms than on the phonological regularities, and better on the written cognates than on the written non-cognates.

The hypothesis of this study was to examine whether the relationship between working memory and previously learned languages (Spanish and English) is stronger in the acquisition of French vocabulary than in the acquisition of Nahuatl vocabulary. To determine this relationship in the present study, Pearson correlation analyses were calculated between the scores on the forward digit span test, the backward digit span test, and the three tasks (See Table 5).

Table 5 shows that the influence of working memory and short-term memory was stronger in the acquisition of French vocabulary than in the acquisition of Nahuatl vocabulary. The correlation analysis showed that regarding phonological regularities and written forms, there was a moderate correlation between the French written forms and the French phonological regularities (*r* = 0.52, *p* < 0.001) and the French combined tasks and the French phonological regularities (*r* = 0.45, *p =* 0.003). Working memory correlated moderately with French combined tasks (*r =* 0.44, *p =* 0.003), but not with phonological regularities in any of the languages studied. Working memory correlated slightly more strongly with the French combined tasks (Task 3) than short-term memory. In contrast, short-term memory, but not working memory, correlated, albeit weakly, with phonological regularities in French (*r =* 0.39, *p* < 0.011). However, it is important to emphasize that there was a strong correlation between the written form (Task 2) and the combined tasks (Task 3) in French and Nahuatl. The strongest component of the combined task (Task 3) was the written form; therefore, Table 5 shows a strong correlation between these two tasks (French *r =* 0.72, *p* < 0.001 and Nahuatl *r =* 0.70, *p* < 0.001). These results indicate that the influence of working memory was stronger when acquiring the vocabulary of a typologically similar language.

Another hypothesis was whether the typology of previously learned languages influences the acquisition of cognates, but not the acquisition of non-cognates in French and Nahuatl. We investigated this hypothesis by conducting repeated measures ANOVAs (See Table 6 and Table 7). We tested the same two-way ANOVA model three times to examine first the cognates, second the non-cognates, and third the task effects (see Table 8).

As can be seen in Table 6, *p* < 0.05 and F > F crit. This shows that there was a significant difference in the acquisition of French and Nahuatl. Taking into account the previous results in Table 5, it can be said with certainty that the acquisition of French vocabulary was stronger in cognates with a high degree of overlap between Spanish and English than in the acquisition of Nahuatl. However, as shown in Table 7, there is no significant difference between the acquisition of French non-cognates and Nahuatl non-cognates. The acquisition of non-cognates is not related at all to cross-linguistic regularities in phonology and written form.

**Figure 1:**
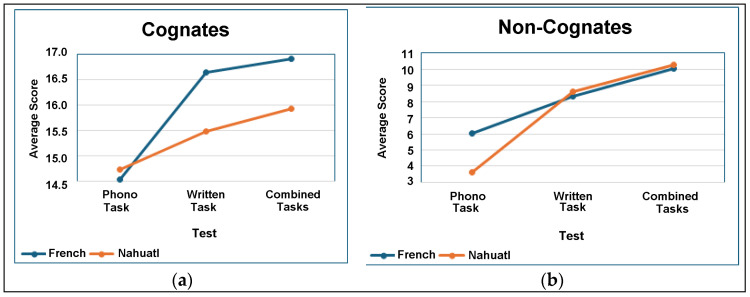
(**a**) Cognates: Tasks 1, 2, and 3 in French and Nahuatl; (**b**) non-cognates: Tasks 1, 2, and 3 in French and Nahuatl.

Table 6 and Table 7 show that in Tasks 1, 2, and 3, *p* < 0.05 and F > F crit. This indicates that there is a significant difference between the tasks. When we combine this with the trends shown in Figure 1a,b, it appears that participants improved their performance across tasks in both cognates and non-cognates. In terms of interaction, properties such as *p* < 0.05 and F > F crit are also fulfilled, although F is a few points above the critical F. 

Finally, we hypothesized that the repetition of the words in Task 1 and Task 2 also had the function of rehearsal and contributed to the acquisition of French and Nahuatl vocabulary. A two-way ANOVA with Task 1, 2 and 3 as the independent variable and the scores in the three tasks as the dependent variable showed that there was a significant main effect of improvement in each task [F (2252) > F critical (84.37 > 3.03), *p* < 0.001] (see Table 8). This finding indicates that scores increased with each task, with the increase and time going in the same direction. The scores increased sequentially and followed the order in which the tests were performed. The pattern is clear: learning performance was improved by rehearsal.

There was also a main effect of the languages studied on the scores of each task F (1252) > F critical (7.77 > 3.87), *p*-value < α (0.005 < 0.05). The results of the ANOVA in Table 8 confirm that there is a significant difference between Nahuatl and French, which shows that French is easier to learn due to the proximity between the languages. The results of the ANOVA, F (2252) < F critical (1.05 < 3.03), and *p*-value > α (0.35 > 0.05) show no significant effect due to the interaction of the two languages studied and the three tasks performed (Tasks 1, 2 and 3). This means that the languages and the tests together have no influence on the students score. Figure 1a,b illustrate the observations from Table 8.

Figure 1a,b show that participants improved over time on all measures between cognates and non-cognates in Tasks 1, 2, and 3. The three tasks were performed at different time points in a sequential order (the phonological task was performed in t0, the written task in t1, and the combined tasks in t2). Figure 1a,b confirm the previous results (Table 8) and show that French cognates are easier to acquire than Nahuatl for this particular group of participants. However, there is no statistical difference between French and Nahuatl with regard to the acquisition of non-cognates. This suggests that the participants made a conscious effort to understand the meaning of the unknown words and that they acquired them in this way [29].

### 3.2. Qualitative Results

Following the testing phases, the participants completed a questionnaire. Their answers are reported in this section: 60% (*n* = 26) of the participants thought that the tasks in Nahuatl were the most difficult to solve, and 47% of them considered that Task 1 (audio) was the most complicated in this language; 60% felt that practicing with the previous tasks (1 and 2) helped them to solve Task 3; 77% stated that Spanish helped them to recognize the meaning of the word; however, 80% indicated that the contribution of this language was less than 40%; 86% stated that their L2 (English) did not contribute at all to solving the tasks.

With regard to the French tasks, 60% thought that it was easier to answer the French tasks than the Nahuatl tasks; 55% thought that the audio tasks were the most difficult to solve; 97% felt that they were able to solve Task 1 because some words sounded like Spanish and others sounded like English; 86% thought that some words in Spanish and English had the same or similar written form in French; 94% mentioned that Spanish helped to solve the French tasks. Similarly, 94% thought that English contributed to solving the tasks; 31% considered that the easiest task was Task 3. They added that answering the previous tasks helped them to identify the meaning of the word with its pronunciation and written form. These results confirmed the findings presented in the previous sections that previously learned languages influence the processing of information in French. 

## 4. Discussion

This study explored the influence of cross-linguistic factors and working memory capacities on vocabulary acquisition, considering the typological distance between Spanish (participants’ native language), English (L2), and the target languages, French and Nahuatl. The results suggest a complex relationship between cross-language factors and working memory abilities, which can either aid or hinder the acquisition of new vocabulary. Three important results were found. First, a correlation analysis indicated a relationship between short-term memory and the recognition of phonological regularities in French. O’Brien et al. found that phonological memory (PM) was associated with low-level vocabulary production in the target language [71]. In this study, French and Nahuatl were languages that the participants had never learned. Thus, this result suggests that learners with low language proficiency may rely more heavily on their short-term memory to acquire vocabulary.

Second, there was a correlation between working memory and the written forms (Task 2). The combined tasks in French and Nahuatl correlated strongly with the written forms. The fact that participants relied more on written tasks could indicate that they were using their working memory to manipulate mental images as they had time to process and store the written information. They may be using their ability to store visual information to coordinate and integrate information from different sources [72]. With regard to this correlation, it was found that the participants’ lexical performance depended on their linguistic background. As participants expressed in the self-reflexive questionnaire, they utilized multiple processes across various levels of linguistic analysis (phonology, morphology, semantics, syntax, and contextual background) in their two languages (Spanish and English) to comprehend unfamiliar written words in French and Nahuatl. This processing may be facilitated by the activation of working memory, which integrates new information with previously stored knowledge from long-term memory [36,60,73]. 

The correlation between the backwards digit span test and French Task 3 was strongest in this study, while it was weaker for Nahuatl. The correlation between successful vocabulary recall and working memory abilities suggests that performance may be predicted in part by processing capacity (i.e., working memory abilities). Engle considers that the role of working memory is the ability to control attention in order to retain or suppress information [72]. Rehearsal, as one of the processes residing in working memory [24], was essential for participants in this study to complete Task 3. Participants made a conscious effort to understand the meaning of the unknown words in the languages studied (French and Nahuatl) in Task 1 and Task 2. These exercises helped participants to discover word meanings and create memory traces for later recall [24], in both French and Nahuatl, although this was clearer in French. This study also emphasizes the importance of rehearsal when learning vocabulary. The repetitive tasks (Tasks 1 and 2) served as an efficient method for practicing, which led to improved performance in Task 3. This highlights the significance of practice and repetition in consolidating new vocabulary in long-term memory, a process supported by working memory [61].

Finally, the analysis revealed that participants recognized similar words (cognates) between Spanish or English and the target languages more accurately than less similar words (non-cognates). This suggests that lexical similarities facilitate word recognition, consistent with previous research on the benefits of typological proximity for vocabulary learning [12]. The relationship between working memory and the category of cognates showed that only in French did working memory have a significantly stronger effect on the category of cognates than on the category of non-cognates. This indicates that the participants transferred relevant knowledge from the typologically closer languages. In the case of French vocabulary, this transfer was motivated by formal similarities between the languages. Furthermore, it appears that the transfer of words may occur from their L1 (Spanish) or L2 (English), depending on which language is typologically closer to French (for example, Spanish *serpiente* and French *serpent* or English *table* and French *table*). This finding is consistent with previous studies [9,43,74,75] showing that working memory supports the acquisition of regularities between typologically close languages. The qualitative data from the participants’ questionnaires further support the quantitative findings. The majority of participants reported that their competence with Spanish and, to a lesser extent, English helped them perform better on the vocabulary exercises with French words. In addition, they mentioned that tasks involving French were generally easier than those involving Nahuatl, attributing this to the phonological and orthographic similarities between Spanish and English. This subjective perception underscores the role of cross-linguistic influence in vocabulary acquisition.

In Nahuatl, on the other hand, this cross-linguistic influence did not occur. This finding is remarkable for the following reasons: First, although there were cognates such as ahuacatl [a ɣwa ‘ka t] in Nahuatl and aguacate [ˌaɣwak’ate] in Spanish, the participants did not transfer this information. In the self-reflection questionnaire, they stated that there were no similarities between Spanish and Nahuatl. This perception limited their intuitions about which regularities they could transfer. Thus, the transferability of what could or could not be transferred between the languages was partly influenced by the participants’ judgement [17]. Secondly, Nahuatl is not typologically close to Spanish. This fact had a negative effect when working memory was used to acquire vocabulary in this language. The interaction of working memory and language acquisition between Nahuatl and Spanish was weak compared to French and Spanish or English. These results support the existing literature, which suggests that typological proximity promotes vocabulary acquisition by facilitating the recognition and retrieval of cognates [5,9].

## 5. Conclusions

The aim of the present study was to investigate the interplay between working memory capacity and cross-linguistic factors in the acquisition of French and Nahuatl vocabulary in Mexican students. Specifically, it delves into the impact of working memory capacity and the typological similarity of previously acquired languages (L1 Spanish and L2 English) to the target languages (French and Nahuatl) on vocabulary acquisition. The results offer valuable insights into the intricate correlation between cognitive functions and linguistic background in L2 learning. The results show that working memory plays an important role in the acquisition of L2 vocabulary, especially in languages that are typologically close. Participants showed a stronger correlation between working memory and vocabulary acquisition in French than in Nahuatl, both in phonological and written tasks, with French performing better on all measures. This suggests that the cognitive load of learning new vocabulary is lower when linguistic similarities are present. Furthermore, this study emphasizes the influence of cross-linguistic factors on the acquisition of cognates compared to non-cognates. The results confirm that cognates that show phonological and orthographic similarities with the learners’ known languages are easier to acquire. This effect is more pronounced in French due to its typological proximity to Spanish and English than in Nahuatl, which is a more distant language. 

In conclusion, this study demonstrates that both working memory and cross-linguistic influence significantly affect L2 vocabulary acquisition. The findings suggest that language learners benefit from the cognitive and linguistic resources derived from their L1 and L2, especially when learning a typologically similar language. These insights have practical implications for language teaching, highlighting the need to leverage learners’ existing linguistic knowledge and to incorporate repetitive, rehearsal-based activities to enhance vocabulary learning. Future research could further explore these dynamics regarding sample size, participant homogeneity, working memory measures, uncontrolled cognitive and affective factors, and the choice of target languages to build on these findings.

Despite the valuable insights gained from this research, it must be acknowledged that there are several limitations to consider. Firstly, the sample size of this study was relatively small, consisting of only 43 Mexican college students. This limited sample may not accurately represent larger populations, thus impacting the generalizability of the findings [70]. In the future, efforts should be made to include a more diverse and larger sample size in order to enhance the robustness of the results.

Another constraint of this study pertains to the homogeneity in linguistic backgrounds among participants. All participants were native speakers of Mexican Spanish with similar levels of English proficiency. This uniformity may not fully capture the variability in working memory and cross-linguistic influence that may be present in learners with different linguistic backgrounds or varying levels of proficiency in their L2 and L3. Including participants from diverse linguistic and educational backgrounds could provide a more thorough understanding of the interactions between working memory and cross-linguistic influence [76].

Furthermore, this study solely relied on the forward and backward digit span tests as measures of working memory capacity. Although these tests are well established [61], they may not encompass all aspects of working memory that are relevant to language learning. Other components, such as the phonological loop or visuospatial sketchpad, could also have significant impacts on vocabulary acquisition [24]. By incorporating a broader range of working memory assessments, a more detailed picture of its influence on language learning could be attained.

Another limitation was the order in which participants were exposed to the tasks in French and Nahuatl. The order of the tasks could have distorted the results. It could be that the tasks in French had a systematic effect on the completion of the tasks in Nahuatl. Future analyses should counterbalance the order of the tasks.

Lastly, the selection of French and Nahuatl as target languages, while strategic in highlighting typological differences, may limit the applicability of the findings to other language pairs. The typological distance between Spanish, English, and these specific languages may not reflect the complexities involved in learning languages with different structural and phonological relationships. Extending the study to include additional language pairs with varying degrees of typological relatedness would help validate and extend the current findings [14].

## Figures and Tables

**Table 1:** Cognates and non-cognates (phonological similarities between French and Spanish or English).

Cognate Categories	French	Spanish	English
**Identical cognates**	Photo [fɔto] Ballon [balɔ̃] Bleu [blø] Collection [kɔlɛksjɔ̃] Docteur [dɔktœʀ] Famille [famij]	Foto [ˈ fo.t̪o] Balón [baˈ lon] Colección [ko.lekˈ sjon] Doctor [dokˈ toɾ] Familia [faˈ milja]	Blue [ blu]
	**French**	**Spanish**	** English **
**Non-identical cognates**	Banane [banan] Bouteille [butɛj] Carotte [kaʀɔt] Dauphin [dofɛ̃] Éléphant [elefɑ̃] Fleur [flœʀ] Hélicoptère[elikɔptɛʀ] Lion [ljɔn] Pantalon [pɑ̃talɔ̃] Serpent [sɛʀpɑ̃] Tomate [tɔmat]	Botella [boˈ t̪e.ʝa] Elefante[eleˈ fante] Flor [‘flor] helicóptero[eliˈ kopteɾo] león [leˈ on] pantalón [pan.taˈ lon] serpiente [seɾˈ pjente] Tomate [toˈ mate]	Banana [bəˈ nænə] Carrot [ˈ kærət] Dolphin [ˈ dɑlfɪn] Elephant [ˈ ɛləfənt] Helicopter[hɛlikɒptə^r^]
	**French**	**Spanish**	**English**
**Non-cognates**	Bouche [buʃ] Coq [[kɔk] Chat [ʃa] Chaise [[ʃɛz] Chien [ʃjɛ̃] Crayón [kʀɛjɔ̃] Fille [fij] Mère [mɛʀ] Père [pɛʀ] Pomme [pɔm] Singe [sɛ̃ʒ] Table [tabl] Tête [tɛt]	Boca [ˈ boka] Gallo [ˈ ɡa.ʝo] Gato [ˈ ɡa.t̪o] Silla [ˈ si.ʝa] Perro [ˈ pe.ro] Lápiz [ˈ la.pis] Hija [ˈ i.xa] Madre [ˈ ma.ð̞ɾe] Manzana [man̟ˈ sa.na] Padre [ˈ pa.ð̞ɾe] Mono [ˈ mo.no] Mesa [ˈ me.sa] Cabeza [kaˈ βesa]	

**Table 2:** Cognates and non-cognates (phonological similarities between Nahuatl and Spanish).

Cognate Categories	Nahuatl	Spanish
**Identical cognates**	Molli [’mo le]	Mole [’mo le]
	**Nahuatl**	**Spanish**
**Non-identical cognates**	Ahuacatl [a ɣwa ’ka t] Axolotl [a ’so lo t] Cahuayo [ka ’ɣwa yo] Chilli [tʃil] Coyotl [ko ’yo t] Élotl [e ’lo t] Mapachtli [ma pa ’tʃ tli] Ocelotl [o ’θe lo t] Petatl [’pe ta t] Pozolatl [po ’so la t] Quetzalli [ke t ’sa li] Texocotl [te ’so ko t] Tlalkakahuatl [tal ka ’ka wa t] Tomatl [’to ma t] Tzictli [t ’sik tli] Xocoatl [’so ko t]	Aguacate [ˌaɣwakˈ ate] Ajolote [a xo ’lo te] Caballo [ka ’βa ʎo] Chile [’tʃi le] Coyote [ko ’yo te] Elote [e ’lo te] Mapache [ma ’pa tʃe] Ocelote [o θe ’lo te] Petate [pe ’ta te] Pozole [po ’θo le] Quetzal [ke t ’θal] Tejocote [te xo ’ko te] Cacahuate [ka ka ’wa te] Tomate [to ’ma te] Chicle [’tʃi kle] Chocolate [tʃo ko ’la te]
	**Nahuatl**	**Spanish**
**Non-cognates**	Cihuatl [’si wa t] Coatl [’ko wa t] Cuixin [ku ’i sin] Hueyi tlati [’we ʎj ’tla ti] Huitzillin [wi t ’si ki] Ixcitl [’ik si t] Maitl [’ma j t] Misto [’mis ton] Pilli [’pi li] Telpochcalli [’tep oks ka li] Tezcatl [’tes kat] Tletl [’ti t] Tochin [’to tʃin]	Niña [’ni ɲa] Serpiente [ser ’pjen te] Gavilán [ga βi ’lan] Abuelo [a ’βwe lo] Colibrí [ko li ’βri] Pie [pje] Mano [’ma no] Gato [’ga to] Niño [’ni ɲo] Escuela [es ’kwe la] Espejo [es ’pe xo] Fuego [’fwe ɣo] Conejo [ko ’ne xo]

**Table 3:** Similar characters.

French	Spanish	English	Nahuatl
Banane Bleu Collection Dauphin Docteur Éléphant Photo Table	Doctor Elefante	Banana Blue Collection[kəlek′shən] Dolphin Doctor Elephant Photo [ˈ foʊtoʊ] Table [ˈ teɪbəl]	
**French**	**Spanish**	**English**	**Nahuatl**
Ballon Bouteille Famille Hélicoptère Lion Pantalon Serpent Tomate	Balón Botella Familia Helicóptero León Pantalón Serpiente Tomate		
**French**	**Spanish**	**English**	**Nahuatl**
	Aguacate Ajolote Caballo Chile Coyote Elote Mapache Mole Ocelote Petate Pozole Quetzal Tejocote Tomate Chocolate		Ahuacatl Axolotl Cahuayo Chilli Coyotl Élotl Mapachtli Molli Ocelotl Petatl Pozolatl Quetzalli Texocotl Tomatl Xocoatl

**Table 4:** Phonological regularities and written form results.

Language			Mean	SD	*n*
French	Phonological regularities	Cognates	14.5	1.96	43
		Non-cognates	6	2.38	43
	Written forms	Cognates	16.7	0.52	43
		Non-cognates	8.3	3.07	43
Nahuatl	Phonological regularities	Cognates	14.7	1.89	43
		Non-cognates	3.6	1.89	43
	Written forms	Cognates	15.5	1.48	43
		Non-cognates	8.6	3.91	43

**Table 5:** Pearson correlation analyses between STM, WM, and the three tasks in French and Nahuatl.

		Phono French	Written French	Comb French	Phono Nahuatl	Written Nahuatl	Comb Nahuatl	STM	WM
Phono French	Correlation	11							
	* p *								
Written French	Correlation	* 0.52	1						
	* p *	<0.001							
Comb French	Correlation	0.45	0.72	1					
	* p *	0.003	<0.001						
Phono Nahuatl	Correlation	0.25	0.21	0.36	1				
	* p *	0.103	0.167	0.017					
Written Nahuatl	Correlation	0.38	0.38	0.34	0.37	1			
	* p *	0.013	0.012	0.028	0.014				
Comb Nahuatl	Correlation	0.26	0.34	0.27	0.28	0.7	1		
	* p *	0.093	0.026	0.08	0.068	<0.001			
STM	Correlation	0.39	0.2	0.36	0.22	0.14	0.06	1	
	* p *	0.011	0.195	0.019	0.157	0.365	0.709		
WM	Correlation	0.24	0.37	0.44	0.16	0.24	0.28	0.37	1
	* p *	0.114	0.013	0.003	0.294	0.12	0.07	0.015	

Note: Phono = phonological regularities; Written = written forms; Combo = combined tasks; STM = short-term memory; WM = working memory. * Pearson correlations *r* values.

**Table 6:** Repeated measures ANOVA for cognates.

Source of Variation	SS	Df	MS	F	*p*-Value	F Crit
French/Nahuatl	26.70	1	26.70	12.28	0.000541	3.87
Tasks 1, 2, 3	152.86	2	76.43	35.15	<0.001	3.03
Interaction	23.82	2	11.91	5.479	0.004685	3.03
Total	751.19	257				

**Table 7:** Repeated measures ANOVA for non-cognates.

Source of Variation	SS	Df	MS	F	*p*-Value	F Crit
French/Nahuatl	25.43	1	25.43	2.85	0.09	3.87
Tasks 1, 2, 3	1284.93	2	642.46	72.14	<0.001	3.03
Interaction	100.83	2	50.41	5.66	0.003932	3.03
Total	3655.15	257				

**Table 8:** Repeated measures ANOVA for cognates and non-cognates.

ANOVA						
Source of Variation	SS	Df	MS	*F*	*p*-Value	F Crit
Languages	106.8062	1	106.8062	7.779576	0.005687	3.878624
Tasks 1, 2, and 3	2316.721	2	1158.36	84.37294	<0.001	3.031629
Interaction	28.93798	2	14.46899	1.053896	0.350108	3.031629
Within	3459.721	252	13.72905			
Total	5912.186	257				

## Data Availability

The data presented in this study are openly available in [Cross-linguistic_datasets] at [https://github.com/Betzigit/crosslinguistic_datasets 28 June 2024].
